# Supplementary material for: Resolving the full spectrum of human genome variation using Linked-Reads
Source: Genome Res. 2019 Apr;29(4):635–45. doi: 10.1101/gr.234443.118 (PMC6442396; doi:10.1101/gr.234443.118)
Supplement: Supplemental Material [file supp_gr.234443.118_Supplemental_longranger-2.2.zip › longranger-2.2.tar.bz2!/lr2.2-public/lib/stan/cmdstan/stan/doc/api/html/categorical__logit_8hpp_source.html]

Stan: src/stan/prob/distributions/multivariate/discrete/categorical\_logit.hpp Source File


|  |  |
| --- | --- |
|  | Stan  2.5.0  probability, sampling & optimization |


- Main Page
- Related Pages
- Namespaces
- Classes
- Files

- File List
- File Members

All Classes Namespaces Files Functions Variables Typedefs Enumerations Enumerator Friends Macros Pages

- src
- stan
- prob
- distributions
- multivariate
- discrete

categorical\_logit.hpp

Go to the documentation of this file.

1 #ifndef STAN\_\_PROB\_\_DISTRIBUTIONS\_\_MULTIVARIATE\_\_DISCRETE\_\_CATEGORICAL\_LOGIT\_LOG\_HPP

2 #define STAN\_\_PROB\_\_DISTRIBUTIONS\_\_MULTIVARIATE\_\_DISCRETE\_\_CATEGORICAL\_LOGIT\_LOG\_HPP

3

4 #include <vector>

5 #include <boost/math/tools/promotion.hpp>

6 #include <stan/math/error\_handling.hpp>

7 #include <stan/math/matrix/log\_softmax.hpp>

8 #include <stan/math/functions/log\_sum\_exp.hpp>

9 #include <stan/math/matrix/log\_sum\_exp.hpp>

10 #include <stan/math/matrix/sum.hpp>

11 #include <stan/prob/traits.hpp>

12

13 namespace stan {

14

15  namespace prob {

16

17  // CategoricalLog(n|theta) [0 < n <= N, theta unconstrained], no checking

18  template <bool propto,

19  typename T\_prob>

20  typename boost::math::tools::promote\_args<T\_prob>::type

21  categorical\_logit\_log(int n,

22  const Eigen::Matrix<T\_prob,Eigen::Dynamic,1>& beta) {

23  static const char\* function = "stan::prob::categorical\_logit\_log(%1%)";

24

25  using stan::math::check\_bounded;

26  using stan::math::check\_finite;

27  using stan::math::log\_sum\_exp;

28

29  double lp = 0.0;

30  check\_bounded(function, n, 1, beta.size(),

31  "categorical outcome out of support",

32  &lp);

33

34  check\_finite(function, beta, "log odds parameter", &lp);

35

36  if (!include\_summand<propto,T\_prob>::value)

37  return 0.0;

38

39  // FIXME: wasteful vs. creating term (n-1) if not vectorized

40  return beta(n-1) - log\_sum\_exp(beta); // == log\_softmax(beta)(n-1);

41  }

42

43  template <typename T\_prob>

44  inline

45  typename boost::math::tools::promote\_args<T\_prob>::type

46  categorical\_logit\_log(int n,

47  const Eigen::Matrix<T\_prob,Eigen::Dynamic,1>& beta) {

48  return categorical\_logit\_log<false>(n,beta);

49  }

50

51  template <bool propto,

52  typename T\_prob>

53  typename boost::math::tools::promote\_args<T\_prob>::type

54  categorical\_logit\_log(const std::vector<int>& ns,

55  const Eigen::Matrix<T\_prob,Eigen::Dynamic,1>& beta) {

56  static const char\* function = "stan::prob::categorical\_logit\_log(%1%)";

57

58  using stan::math::check\_bounded;

59  using stan::math::check\_finite;

60  using stan::math::log\_softmax;

61  using stan::math::sum;

62

63  double lp = 0.0;

64  for (size\_t k = 0; k < ns.size(); ++k)

65  check\_bounded(function, ns[k], 1, beta.size(),

66  "categorical outcome out of support",

67  &lp);

68

69  check\_finite(function, beta, "log odds parameter", &lp);

70

71  if (!include\_summand<propto,T\_prob>::value)

72  return 0.0;

73

74  if (ns.size() == 0)

75  return 0.0;

76

77  Eigen::Matrix<T\_prob,Eigen::Dynamic,1> log\_softmax\_beta

78  = log\_softmax(beta);

79

80  // FIXME: replace with more efficient sum()

81  Eigen::Matrix<typename boost::math::tools::promote\_args<T\_prob>::type,

82  Eigen::Dynamic,1> results(ns.size());

83  for (size\_t i = 0; i < ns.size(); ++i)

84  results[i] = log\_softmax\_beta(ns[i] - 1);

85  return sum(results);

86  }

87

88  template <typename T\_prob>

89  inline

90  typename boost::math::tools::promote\_args<T\_prob>::type

91  categorical\_logit\_log(const std::vector<int>& ns,

92  const Eigen::Matrix<T\_prob,Eigen::Dynamic,1>& beta) {

93  return categorical\_logit\_log<false>(ns,beta);

94  }

95

96

97  }

98 }

99 #endif

log\_sum\_exp.hpp

stan::math::check\_bounded

bool check\_bounded(const char \*function, const T\_y &y, const T\_low &low, const T\_high &high, const char \*name, T\_result \*result)

**Definition:** check\_bounded.hpp:79

stan::agrad::log\_sum\_exp

fvar< T > log\_sum\_exp(const fvar< T > &x1, const fvar< T > &x2)

**Definition:** log\_sum\_exp.hpp:15

traits.hpp

stan::math::log\_sum\_exp

boost::math::tools::promote\_args< T1, T2 >::type log\_sum\_exp(const T2 &a, const T1 &b)

Calculates the log sum of exponetials without overflow.

**Definition:** log\_sum\_exp.hpp:49

stan::prob::categorical\_logit\_log

boost::math::tools::promote\_args< T\_prob >::type categorical\_logit\_log(int n, const Eigen::Matrix< T\_prob, Eigen::Dynamic, 1 > &beta)

**Definition:** categorical\_logit.hpp:21

error\_handling.hpp

stan::math::check\_finite

bool check\_finite(const char \*function, const T\_y &y, const char \*name, T\_result \*result)

Checks if the variable y is finite.

**Definition:** check\_finite.hpp:52

stan::agrad::log\_softmax

Eigen::Matrix< fvar< T >, Eigen::Dynamic, 1 > log\_softmax(const Eigen::Matrix< fvar< T >, Eigen::Dynamic, 1 > &alpha)

**Definition:** log\_softmax.hpp:16

stan::agrad::sum

fvar< T > sum(const Eigen::Matrix< fvar< T >, R, C > &m)

**Definition:** sum.hpp:14

stan::prob::include\_summand

Template metaprogram to calculate whether a summand needs to be included in a proportional (log) prob...

**Definition:** traits.hpp:35

log\_softmax.hpp

stan::math::log\_softmax

Eigen::Matrix< T, Eigen::Dynamic, 1 > log\_softmax(const Eigen::Matrix< T, Eigen::Dynamic, 1 > &v)

Return the natural logarithm of the softmax of the specified vector.

**Definition:** log\_softmax.hpp:44

stan::math::sum

double sum(std::vector< double > &x)

**Definition:** sum.hpp:10

log\_sum\_exp.hpp

sum.hpp


---

[ Stan Home Page ]

*© 2011–2014,
Stan Development Team.*
